# Implementation of social needs screening for minoritized patients newly diagnosed with breast cancer: a mixed methods evaluation in a pragmatic patient navigation trial

**DOI:** 10.1186/s12913-024-11213-7

**Published:** 2024-07-09

**Authors:** Stephenie C. Lemon, Amy M. LeClair, Erika Christenson, Deborah Amburgey, Madyson FitzGerald, Howard Cabral, Chris Lloyd-Travaglini, Cheryl R. Clark, Feng Qing Wang, Joellen Ross, Ellen Ohrenberger, Jennifer S. Haas, Karen N. Freund, Tracy A. Battaglia, the TRIP Consortium [representative Tracy A. Battaglia, Ted A. James, Jessica Shenkel, Susan T. Gershman, Mark Kennedy, Anne Levine, Erica T. Warner, Naomi Y. Ko, Debi Amburgey, Julia Vance, Victoria Xiao, Tony Zhao, Howard J. Cabral, Clara Chen, Christine Lloyd-Travaglini, Julianne Dugas, Magnolia Contreras, Rachel A. Freedman, Karen Burns White, Christine Gunn, Beverly Moy, Caylin Marotta, Amy J Wint, Susan K. Parsons, Serena Rajabiun

**Affiliations:** 1 Division of Preventive and Behavioral Medicine, UMass Chan Medical School, 55 Lake Avenue North, Worcester, MA 01655 USA; 2 Tufts Medical Center, Boston, MA USA; 3 Boston Medical Center, Boston, MA USA; 4 Boston University School of Public Health, Boston, MA USA; 5 Brigham and Women’s Hospital, Boston, MA USA; 6 Beth Israel Deaconess Medical Center, Boston, MA USA; 7 Massachusetts General Hospital, Boston, MA USA

**Keywords:** Patient navigation, Breast cancer, Social needs, Implementation, Mixed methods

## Abstract

**Background:**

Social needs inhibit receipt of timely medical care. Social needs screening is a vital part of comprehensive cancer care, and patient navigators are well-positioned to screen for and address social needs. This mixed methods project describes social needs screening implementation in a prospective pragmatic patient navigation intervention trial for minoritized women newly diagnosed with breast cancer.

**Methods:**

Translating Research Into Practice (TRIP) was conducted at five cancer care sites in Boston, MA from 2018 to 2022. The patient navigation intervention protocol included completion of a social needs screening survey covering 9 domains (e.g., food, transportation) within 90 days of intake. We estimated the proportion of patients who received a social needs screening within 90 days of navigation intake. A multivariable log binomial regression model estimated the adjusted rate ratios (aRR) and 95% confidence intervals (CI) of patient socio-demographic characteristics and screening delivery. Key informant interviews with navigators (n = 8) and patients (n = 21) assessed screening acceptability and factors that facilitate and impede implementation. Using a convergent, parallel mixed methods approach, findings from each data source were integrated to interpret study results.

**Results:**

Patients’ (n = 588) mean age was 59 (SD = 13); 45% were non-Hispanic Black and 27% were Hispanic. Sixty-nine percent of patients in the navigators’ caseloads received social needs screening. Patients of non-Hispanic Black race/ethnicity (aRR = 1.25; 95% CI = 1.06–1.48) and those with Medicare insurance (aRR = 1.13; 95% CI = 1.04–1.23) were more likely to be screened. Screening was universally acceptable to navigators and generally acceptable to patients. Systems-based supports for improving implementation were identified.

**Conclusions:**

Social needs screening was acceptable, yet with modest implementation. Continued systems-based efforts to integrate social needs screening in medical care are needed.

**Supplementary Information:**

The online version contains supplementary material available at 10.1186/s12913-024-11213-7.

Contributions to the literature

- This study observed high degrees of acceptability of social needs screening delivery by patient navigators and high degree of acceptability of social needs screening by patients in the context of treatment for breast cancer among newly diagnosed, minoritized women.
- This study observed that despite resources and training, screening for social needs by patient navigators in the context of routine cancer is delivered to modest percentage (69%) of eligible patients.
- This study identified systems-level interventions that could enhance performance of social needs screening by patient navigators in breast cancer care.

## Background

Despite decades of progress in breast cancer treatment, disparities in outcomes persist [1, 2]. Women diagnosed with breast cancer who are Black, Hispanic, speak a primary language other than English, and/or who have inadequate insurance experience high rates of delays in care, contributing to increased morbidity and mortality [2–5].

Unmet social needs such as housing insecurity, food insecurity and lack of transportation are barriers to timely cancer care. An estimated 20% of patients with cancer in the United States experience at least one unmet social need, with higher rates among individuals of Black race, Hispanic ethnicity and/or low socioeconomic status [6–8]. Calls for the integration of systematic social needs screening with appropriate follow-up into medical care in general and cancer care specifically have accelerated as a means to ameliorate barriers and improve equitable health outcomes [9–13].

However, systematic social needs screening remains aspirational, as it not a routine part of cancer care in most delivery sites [14]. Little research has assessed how to successfully implement social needs screening in the context of cancer care. Patient navigators are vital members of cancer care teams who are potentially well-suited to deliver social needs screenings and work with patients to address identified needs. Patient navigation is an evidence-based approach for reducing cancer-related disparities, now considered standard of care [15–17]. Patient navigators are typically responsible for working with patients, especially those at high risk for disparities, to identify and overcome barriers to receipt of timely, high quality cancer care [18, 19]. Thus, screening for and addressing social needs among at-risk patients is consistent with the goals and values of patient navigation.

The purpose of this study is to gain an in-depth understanding of the implementation of social needs screening by patient navigators for newly diagnosed patients with breast cancer from minoritized backgrounds in a pragmatic trial. Specifically, the aims are to quantify the percentage of eligible patients who received social needs screening and patients factors related to delivery of social needs screening and to describe the acceptability of social needs screening and factors that could improve the implementation from the patient navigator and patient perspectives.

## Methods

### The Translating Research Into Practice (TRIP) study

The aim of this mixed methods project was to describe social needs screening implementation in a pragmatic patient navigation intervention trial for minoritized women newly diagnosed with breast cancer. Data are from the TRIP study [20]. Briefly, TRIP used a community engaged approach, with academic partners from four Clinical and Translational Science Institutes in Massachusetts partnering with the Boston Breast Cancer Equity Coalition to address disparities in breast cancer outcomes. TRIP was a Type 1 hybrid effectiveness-implementation trial [21] that utilized a cluster-randomized, stepped wedge design [22, 23], and was conducted in five cancer care centers in Boston, MA between 2018 to 2022.

TRIP aimed to improve receipt of timely, quality breast cancer care through implementation of an integrated, evidence-based patient navigation intervention. The TRIP intervention was integrated as standard practice at the participating sites, each of which had existing breast cancer patient navigation programs [24]. The intervention entailed patient navigators using a standardized, 11-step protocol [25], which included social needs screening and referrals, and was grounded in the principles of patient navigation and care management [26]. The work of the patient navigators was supported by a project-specific shared REDCap registry that guided their caseload through each of the 11-steps. Initial training in administering the TRIP protocol involved in-person and online training in the protocol, use of the registry and use of the standardized social needs screening tools and referral platforms. Quarterly case-based navigator network meetings created a learning collaborative for navigators to share best practices in protocol implementation. In response to navigator’s request for support in asking sensitive questions, a one-hour training in principles of empathic inquiry and using patient-centered approaches to screening adapted from an existing program that integrates basic principles of motivational interviewing and trauma-informed care [27] was conducted after the project began.

### Social needs screening delivery

#### Study design

Data were from the larger TRIP study trial, which utilized a prospective, stepped-wedge design. For this analysis, given the focus on delivery of social needs screening at a single time point, we used a cross-sectional analysis to quantify the delivery of social needs screening per study protocol, defined as completion within 90 days of intake.

#### Participants

To be included in the study population, women had to have a new breast cancer diagnosis between June, 2018 and August, 2021, be at least 18 years of age, and live within the Great Boston area and meet at least one of the following criteria: Black race and/or Hispanic ethnicity, speak a primary language other than English, have public insurance or be uninsured at the time of diagnosis and be part of the navigators’ caseload, as indicated by being entered into the project registry.

#### Measures

##### Social needs screening

Social needs screening was a core component of the TRIP intervention. A standardized screening tool was used at each site with a navigation protocol requiring administration at intake and three-month follow-up intervals. A study-specific tool was adapted from published web-based screening tools and platforms (i.e., Findhelp [28], THRIVE [29]. The tool assessed 9 social need domains: housing insecurity, food insecurity, transportation, paying for treatment, paying for basic utilities, employment, education, family caregiving and legal issues. hese data were prospectively entered into either the site’s Epic Electronic Health Record or the Aunt Bertha/FindHelp platform. For this paper, the intake social needs screening was assessed. The study protocol required eligible patients to receive a social needs screening within 90 days of intake. Data were abstracted from Epic or the Aunt Bertha/FindHelp platform for analysis.

##### Patient socio-demographic characteristics

Patient socio-demographic characteristics were obtained from each site’s Cancer Registry and electronic health record. Characteristics assessed included patient age, race/ethnicity, preferred language and insurance status as indicated at time of diagnosis.

#### Statistical analysis

Frequency distributions quantified delivery of a social needs screening. Bivariate analyses were conducted to assess the frequency of screening for social needs according to patient socio-demographic characteristics. Log binomial regression models were computed to estimate the rate ratio of each patient socio-demographic characteristic with delivery of social needs screening, accounting for clustering within cancer care site. Single variable models and a multivariable model which included all patient socio-demographic characteristics were computed. A two-sided p-value of < 0.05 was used as a threshold of statistical significance. Analyses were performed using SAS software version 9.4 (SAS Institute Inc., Cary, NC).

### Acceptability of social needs screening by patient navigators & patients

#### Study design

Key informant interviews were conducted with the participating patient navigators and a sample of patients. Patient navigator interviews were conducted 27 months after the TRIP protocol was first introduced at the respective project site. Patient interviews were conducted between February and April of 2022. These one-time interviews were conducted by trained project staff over the telephone. The interviews included questions about all of the components of the TRIP intervention: This analysis focuses on the social needs screening component.

#### Participants

*Patient navigators* who participated in the interviews were individuals who were employed by the partnering cancer care centers and working on the TRIP protocol at the time of the respective interviews. All interviews were audio recorded and followed a semi-structured interview guide. Patient navigators were compensated $50. A total of eight patient navigators were eligible and eight participated. Two of the eight were nurse navigators and six were patient navigators without clinical backgrounds.

*Patients* who participated in the interviews: 1) were a patient during the last 6 month of study enrollment (5/1/2021–11/30/2021), 2) spoke English as their preferred language, and 3) had at least one completed social needs assessment. An opt-out letter was sent to all identified patients; patients who did not opt-out received a call from study staff one week after the mailing with up to two follow-up calls. All interviews conducted over the phone by trained study team members, were audio recorded, and followed a semi-structured interview guide. Patients were compensated $25 for their participation. A total of 21 patients were interviewed, with representation from each site.

#### Interview guides

Semi-structured interview guides were developed by the project team and are available in a supplementary file. The guide for patient navigators was designed to understand their experiences and perspectives related to the TRIP navigation protocol and their experiences in the project overall. Open-ended questions with prompts were designed to elicit perspectives related to administering the social needs screening to patients, with an emphasis on understanding acceptability of conducting the screening and barriers and facilitators to implementing the screening tool. [30] The guide for patients was designed to understand their experiences and perspectives related to receiving patient navigation services. Specific questions with prompts were designed to elicit perspectives related to being administered the social needs screening and any subsequent referrals or other follow-up received based on the screening, with an emphasis on acceptability and associated barriers and facilitators [30].

#### Qualitative analysis

The interview recordings were transcribed by Datagain. Transcripts were de-identified and reviewed against audio recordings for accuracy. Rapid qualitative analysis was used [31] to synthesize interview findings. Rapid methods are increasingly used in implementation science and have demonstrated a high degree of concordance with traditional qualitative analytic techniques. Rapid qualitative analysis is especially useful when analyzing action-oriented, time-sensitive qualitative research [31, 32]. Following a previously established approach [33, 34], TRIP team members created a template of domains based on the interview guide that was used to summarize each interview. Two lead team members summarized the first interview independently and discussed their findings to develop a summary matrix and ensure consistency and completeness. The remaining transcripts were divided among four other team members for categorizing (DA, VX, MA, TZ). Team members reviewed each other’s completed summary templates, and made refinements to the template and process as needed. The two team leads with extensive experience in qualitative research (AML, EC) served as the secondary reviewer for each transcript. The summary templates were then combined to create a matrix of responses across all interviews. From this matrix, the domains were reviewed and synthesized into resulting content areas, themes, and representative quotations. Patient navigator and patient interviews were analyzed separately. Results were then compared across interview type to determine commonalities and differences and draw overall inferences.

### Mixed methods data integration

This study used a convergent parallel mixed methods approach to integrate and interpret the results across the three study sources: quantitative social screenings delivery data, patient navigator interviews and patient interviews. In this approach, data are collected concurrently, analyzed separately and given equal priority. Data are then integrated/compared to interpret study results. After analyses were completed, the data from all sources were compared and integrated. Through a series of meetings, the study team examined the relationship between the data from the three sources (quantitative, 2 qualitative sources) to create an explanatory model for the modest findings related to social needs screening delivery.

## Results

### TRIP study sample

The TRIP intervention study sample included 588 women who received a navigator intake assessment. A description of the sample is presented in Table 1. One-third was aged 65 or older. Almost half of the sample (47%) were of African American or Black race and more than one-fourth (27%) were Hispanic. Almost half primarily spoke a language other than English with over 20 languages reported, and had public insurance.

**Table 1:** Social needs screening delivery at intake in the total patient sample and by patient socio-demographic characteristics, *the TRIP study,* 2018–2022 (n = 588)

Characteristic	Total eligible patients n (%)	Received intake social needs screening, %
Total sample	588	69%
Age		
< 50	147 (25%)	69%
50–64	247 (42%)	68%
65 and older	194 (33%)	70%
Race/Ethnicity		
Non-Hispanic White	72 (12%)	49%
Non-Hispanic Black	265 (45%)	77%
Non-Hispanic Asian	75 (13%)	76%
Hispanic	161 (27%)	66%
Other Race	15 (3%)	40%
Preferred language		
English speaking	299 (51%)	66%
Non-English speaking	289 (49%)	72%
Insurance		
Private/Commercial	214 (36%)	69%
Medicare	93 (16%)	78%
Medicaid or Uninsured	281 (48%)	66%
Residence		
Boston	359 (61%)	74%
Greater Boston area	229 (39%)	62%

### Social needs screening delivery

The overall percentage of patients screened for social needs at intake and screening rates according to patient socio-demographic characteristics are presented in Table 1. Overall, 69% of patients in the navigators’ caseloads received this screening. Differences in rate of screening by site were observed, ranging from 40 to 100%. Results were similar across the bivariate and multivariable models (Table 2). In the multivariable model, patients of non-Hispanic Black race/ethnicity were more like to receive social needs screening (aRR = 1.25; 95% CI = 1.06–1.48) compared to patients who were non-Hispanic white. Patients with Medicare insurance were more likely to be screened (aRR = 1.13; 95% CI = 1.04–1.23), compared to patients with private insurance.

**Table 2:** Bivariate and multivariable log binomial models^a^ estimating the rate ratio (RR) and 95% confidence intervals (CI) of patient socio-demographic characteristics with social needs screening delivery, *the TRIP study,* 2018–2022 (n = 588)

Characteristic	Bivariate log binomial models RR (95%)	Multivariable log binomial model RR (95% CI)
Age		
< 50	Ref	Ref
50–64	0.96 (0.90–1.02)	0.94 (0.89–1.01)
65 and older	1.00 (0.91–1.10)	0.93 (0.85–1.03)
Race/Ethnicity		
Non-Hispanic White	Ref	Ref
Non-Hispanic Black	**1.20 (1.06–1.37)**	**1.25 (1.06–1.48)**
Non-Hispanic Asian	1.13 (0.77–1.64)	1.06 (0.68–1.65)
Hispanic	1.05 (0.88–1.25)	1.02 (0.84–1.25)
Other Race	**0.55 (0.43–0.73)**	**0.56 (0.41–0.75)**
Preferred language		
English speaking	Ref	Ref
Non-English speaking	1.02 (0.93–1.12)	1.14 (0.96–1.34)
Insurance		
Private/Commercial	Ref	Ref
Medicare	**1.11 (1.03, 1.21)**	**1.13 (1.04–1.23)**
Medicaid or Uninsured	0.96 (0.90–1.04)	1.03 (0.97–1.09)
Residence		
Boston	Ref	Ref
Greater Boston area	0.91 (0.82–1.02)[p	0.97 (0.87–1.08)

^a^All models account for clustering within cancer care site; Multivariable model adjusts for all patient characteristics

### Patient navigator and patient acceptability of social needs screening

Table 3 describes acceptability-related themes and representative quotations from patient navigators and patients. Patient navigators found screening patients for social needs to be unanimously acceptable. They strongly believed in the purpose and goals of screening patients for social needs in the context of their broader cancer care. The patient navigators additionally perceived that screening for social needs aligned with their professional values and understanding of their roles as patient navigators. The navigation protocol and tools for conducting screenings were found to be acceptable and allowed for them to approach their work in a more standardized manner.

**Table 3:** Acceptability of social needs screening among patient navigators (n = 8) and patients (n = 21), *the TRIP study,* 2018–2022

Theme	Representative quotations
Patient navigators believed in purpose/goals of screening and found it to be consistent with their views on their roles as navigators	“I think that and hope that I will continue to be asking these really relevant questions to my patients, to find out what their needs are, and how we can help them to get better access to care here at our own institution, let alone, TRIP helping them with anything else, but to make sure, that they are able to continue with the rest of their lives, while they’re getting their breast cancer treatment because so many of them end up with taking time off from work or other things.” “The social needs assessments for sure. I think that's a huge reason why a lot of patients have distrust in health care systems. The patients think that the health care system, doctors only really care about doing the surgery or doing the treatment and getting paid for it, whether or not that's truly beneficial to the patient, a lot of people distrust the system. I think trying to let them know that, ‘We're here to help. We want to treat all of you, not just a little bit of you with cancer, figuratively.’ I think trying to work on that and share that with patients I think would be really important. And social needs assessments help with that.”
Patient navigators found the standardized protocol and tools for implementing social needs screenings to be acceptable	"I like TRIP because we get to talk to all our patients and ask them what they actually need, opposed to,’All right. This is what’s going to happen, and this is your appointment, and now you have cancer and that’s it.’ I like the fact that we ask the patient, ‘Do you need anything from us that would make this little trip that you’re having easier?’" "I was just very pleased that that this was incorporated into the practice, because I believe that oftentimes if you're not intentionally looking for or trying to identify these issues, then they may not otherwise rise to the surface. So, I think a win is that we likely identified more patients who needed,social needs support, and ultimately, that that made their care their experience all that much more richer."
Patients felt comfortable being asked sensitive questions about their social needs, despite perceived stigma	“It made me feel good… I'm not like it's not like I'm on welfare and even if I've been on welfare before, but they didn't treat me, how can I say, like I was an inferior person. They didn't make me feel bad at all. So, I was comfortable with it.” "I'm so glad they asked me about this question because sometimes you, we don't want to share things like that…to be in that situation. Sometimes it can be stressful or you'd be ashamed, but I really was happy they asked me all this…" "I felt comfortable because they were the ones who brought it up in the first place. So, it was helpful.” "They definitely made me feel very comfortable with speaking with them, with especially with my needs, so that I can feel more at ease
Patients felt supported when asked about their social needs	“I felt really taken care of. They know what the needs are, they knew what the patient is looking for, things that you don't think about right then. You're worried about your health, how you're going to manage all that. So, knowing this that there is help out there in different areas really, it helps a lot, it takes the pressure off worrying about this thing.” “They really wanted to help me in any area that I needed help with and from day one, I remember the [navigator] started the conversation. If I need anything, I can come forward and let them know if I'm comfortable with my home, my situation, this is the day I learned this, my condition. So, I really felt supported and they tried to understand, they tried to read my mind”

Patients also generally found being screened for social needs to be acceptable. Many patients reported that they felt comfortable being screened for social needs. They additionally reported that being screened for social needs and receiving assistance to address them made them feel more supported during their cancer care. Some patients noted perceptions of stigma around being asked sensitive questions and the emotional impact they experienced in response to having social needs. However, these individuals also noted that the patient navigators were non-judgmental and supportive in their approach, which influenced their willingness to answer social needs questions.

### Factors could improve social needs screening implementation

Table 4 presents barriers and facilitators to social needs screening. Four themes were identified that could improve social needs screening implementation. First, patient navigators expressed that being well-integrated into the larger cancer care team was an important facilitator of establishing relationships with patients. Several patient navigators reported that being in close physical proximity to the physicians and nurses increased these providers’ awareness of and trust in the patient navigators, which resulted in direct provider introductions of patients to the navigators. These “warm handoffs” facilitated more positive navigator-patient interactions, including patient acceptability of social needs screening.

**Table 4:** Identified challenges and supports needed for social needs screening by patient navigators (n = 8) and patients (n = 21), *the TRIP study,* 2018–2022

Theme	Representative quotations
Integrating patient navigators as part of the care team	"Being on site becomes key. You know, they [members of the cancer care team] see you. They let you know when your patient is in. They let you know if they have a new referral or they think someone could use services. Being present is very important." "Once…the provider, or the nurse, or the doctor trusts you more, then they will directly come to you after meeting with the patient." “I work with the team very closely. And then, once I got more support from the team like the nurse who I worked with the screening, and then a social worker I work with some patient for the immediately, I just got a lot of support you know, from the care team. And then I would say, you know, that's most important for me to, working on those projects, because, you know, I'm the only single person, but the work has to relate to the whole team. So, if I don't have the teamwork, I cannot be done, … I cannot provide the quality service support for the patient
Meeting patients and conducting social needs assessments onsite during clinic visits, rather than over the phone	“"I would like to do [the social needs screening] in person when they come in for the first visit in the clinic. That's really helpful compared with the phone,…, to let the patient know,, I'm here, what's my role, and what kind of support we can provide. It makes the patient feel more supported." “I did have some difficulty in being able to meet the patients face to face [during COVID], which was something challenging because I was not able to see them through the phone. And some patients that have trouble understanding my role. So, I had to find ways to better explain my role in why I was calling them and just everything in general. Why I was reaching out to them.” “Sometimes it was challenging specifically when you were trying to do it over the phone because you [are[] asking very personal questions and some patient understandably [are] hesitant to answer those questions. At times they were not kind of willing or they did not feel kind of comfortable to share the information, so they would deny all the answers decline all the answers. So, I mean, once in a while you would have somebody who did not want to engage.” “First of all, whenever possible I try to meet a patient in person because that way they know me,, when I call them, they already are able to identify who is calling them. And she was struggling with her housing because she was renting one, basically a small apartment in her brother's house. And I was able to make sure that she has access to everything she needed.”
Patient navigator comfort screening and assisting patients who speak different languages than they do	[It is] “difficult at times to know whether patients were being honest with us as far as the social needs assessment goes because I’m a white woman, and I don’t speak other languages… So, if I’m dealing with Chinese and/or Asian, and Spanish-speaking patients, or Russian-speaking [patients], whatever it is, I don’t know, are they really telling me everything that’s going on.” "Sometimes for follow up regarding social needs referral, it's a little bit hard, you know, because some, some patients especially for the senior patient who doesn't speak English, they don't have email, they always request to mail to their house, but once I call them to follow up to see if they received, you know, the results it’s always, ‘No, I haven't received that, and then just mail it again.’”
Empathic approaches for patient navigators to conduct screenings	"Once you have more experience and then you know how to provide the patient directly the support…when I share with the patients that I have been seeing like more than 10 patients like your situation, and then we provide the TRIP support, and then they help [on needs] one, two, three, A,B,C,D something like that, it feels like the patient can get more l comfortable to using the resource. And then to use the patient navigation service to get the care, to get more support." Just in talking to patients about, like, "Where do you live, do you feel safe there? Are you having any problems with anything there?" And then just saying, "Do you have lead paint, that's a terrible thing to have", just kind of commenting on things or, "I had lead paint," or something like this, I just kind of make it a little bit more personal... so they feel like they're not talking to somebody that doesn't have some of the same problems that they have.”

Second, patient navigators indicated a strong preference for first meeting and conducting intake social needs screenings with patient’s in-person, rather than over the phone. This was perceived to help establish relationships and patient comfort level with the patient navigator, which set the stage for more fruitful phone-based follow-up conversations. Third, some patient navigators reported low levels of comfort screening and more generally serving patients who spoke different languages than they did. Lastly, patient navigators described that using empathic approaches improved their self-efficacy in delivering social needs screening. Such approaches, which allow navigators to approach screening more conversationally rather than simply reading a survey, allowed navigators to connect better with patients, which was perceived to increase patient comfort level as well.

## Discussion

This mixed methods study examined the implementation of social needs screening among newly diagnosed breast cancer patients at high risk for poor outcomes by patient navigators in a pragmatic trial. Social needs screening was universally acceptable by patient navigators, with patients indicating a high degree of acceptability. However, despite a standardized protocol and patient navigators with effort supported to conduct this screening, patient navigators delivered systematic social needs screening to less than 70% of eligible patients. Overall, approximately seven out of ten eligible patients received an intake social needs screening. These findings mirror results of similar efforts to implement social needs screening in primary care, emergency department, and oncology settings [29, 35–37], with low or modest implementation despite supports to providers to conduct screenings.

Similar to other studies [38], our findings indicate that social needs screening is highly acceptable to providers. This investigation adds to the current literature by focusing on social needs screening in cancer care by patient navigators. Many cancer care sites employ patient navigators as part of the care team [24], and social needs screening is consistent with established goals and values of patient navigation [18]. In this study, navigators universally perceived that addressing patient social needs was important and consistent with their responsibilities. Furthermore, in the context of this research study, patient navigators appreciated the structure provided by the standardized screening tool and referral system.

We identified important differences in screening by patient socio-demographic characteristics. Non-Hispanic Black women had the highest likelihood of screening, compared to other race/ethnicities. Patients with Medicare were more likely to be screened, even when controlling for age. We explored the possibility that individuals with disabilities who receive Medicare, typically indicated by being under age 65, were more likely to be screened. We conducted an ad hoc exploratory analysis that included an interaction between insurance status and age < 65 and over 65) to determine if there was differential response across insurance categories for those younger than 65 compared to those 65 and over. We found no such differential response, hence indicating that higher degree of screening among those with Medicare insurance was not due to disability (indicated by younger age) status. Reasons for differences in screening by patient socio-demographic characteristics observed in this study require further investigation for potentially intervenable factors including implicit bias of the navigators about who may have social needs or which patients are comfortable with social needs inquiry.

Despite universal acceptability, site-level differences in screening rates were observed. The site with the lowest screening rate (40%) experienced navigator staffing shortages prior to [24] and on and off throughout the course of the project. Likewise, the two sites with the highest screening rates (97% and 100%) had established navigation programs that were engrained within the cancer care teams [24]. Previous studies have found that patient navigators can be pulled into other support tasks not associated with navigation [39], which was reported anecdotally by navigators in this project in the context of the COVID-19 pandemic. Research assessing the implementation of social needs screening in primary care settings shows that when systems are in place to support social needs screening, it can be implemented widely [29], and is acceptable to both providers, patients, and caregivers [40, 41]. Our findings further highlight the need for cancer care sites to invest in systems that support consistency of navigation services.

Also similar to previous studies [38], our findings indicate that social needs screening is acceptable to patients. Patients in this study acknowledged the potential stigma and emotional distress that could be associated with sharing personal information on social needs with a care provider. However, they for the most part expressed comfort being asked and sharing their personal circumstances and needs with the patient navigators, because they felt like the navigators were acting in their best interest. Non-clinical patient navigators, who share similar socio-demographic backgrounds and lived experiences to the patient populations they serve, may be particularly well-positioned to establish the requisite comfort and trust in the provider-patient relationship to increase patient acceptability, and willingness to participate in the social needs screening and referral process [42]. This would be consistent with other studies that demonstrate a positive effect on outcomes when there is race and language concordance among patients and navigators [43].

Our qualitative findings additionally highlight systems-level factors that could improve social need screening implementation. Patient navigators simultaneously noted that integration into the broader cancer care team, facilitated by physical proximity, and the ability to meet and conduct initial social needs screenings with patients onsite during a clinic visit, were important for setting the foundation of comfortable, trusting relationships with patients. Previous studies have observed the importance of integrating patient navigators into the healthcare team to achieving robust and successful patient navigation programs [44], and trust building is a core principle of patient navigation [19]. Allocation of appropriate physical space in close proximity to the larger care team has been identified as a best practice for supporting non-clinical health worker integration into care teams [45]. However, patient navigators can be physically siloed from the care team, relegated to non-clinical space or telephone only contact with patients. Presence afforded by physical proximity may help legitimize patient navigators as part of the care team.

Some navigators expressed less comfort screening patients who spoke different languages than they did and concerns about patient comfort with disclosing their needs, even though no differences in delivery of screening by patient primary language were observed. We were unable to assess patient perspectives on this, as the patient interviewers were limited to English speakers. However, a breadth of prior research indicates that language and cultural concordance are associated with more trusting patient-provider relationships [46]. Further investments on the part of health care systems to identify and employ patient navigators with the ability to speak the languages that better reflect the patients they serve may be needed to increase screening, and referrals for social needs.

Patient navigators in this study reported that the ability to approach social needs screening in a manner that was conversational and engaging was key to their comfort-level. The TRIP project incorporated training in empathic inquiry based on early feedback from the patient navigators that they were uncomfortable simply reading a checklist-based survey to patients on sensitive topics and that they felt patients were less likely to answer honestly with this type of approach. The goal is to support health professionals to deliver social needs screening in a manner that is non-judgmental, collaborative, and compassionate, and uses a conversational approach. Our qualitative findings suggest that this approach was well-received and improved navigator self-efficacy. Cancer care sites should invest in communications skills training to support patient-centered care and potentially increase the impact of their investments in patient navigation programs.

Study strengths include the diverse patient sample and the mixed methods approach. However, several limitations must be acknowledged. The study focused on social needs screening and did not assess referrals for identified needs or follow-up, which are essential to address social needs and warrant future investigation. Reasons for the observed differences in social needs screening receipt by race and ethnicity require further investigation for potentially intervenable factors. However, given the convergent parallel design, we were not able to explore these results with our qualitative methods. We also could interpret findings related to women of other race because the sample size is small and the group is very heterogenous. Generalizability is limited as the study occurred in Boston, MA, which has well-established cancer care sites that do not reflect care provision in other regions. The patient interviews were limited to individuals who speak English, so patient barriers related to linguistic equity could not be assessed.

Social needs screening is becoming a standard of care not only for cancer care but for all clinical practice. The Centers for Medicare and Medicaid (CMS) have included screening for health related social needs as one of five priority areas in their framework for achieving health equity [13]. Rules on social needs screening is already part of payment models for post-acute care, and currently 32 states require social needs screening as part of their payment models within their Medicaid program [47]. The CMS 2024 Medicare Physician Fee Schedule (PFS) Final Rule,1 approved in November 2023, now allows patient navigation services and screening for social needs to be reimbursed under new codes. With this rule in place, practices nationwide will need to address findings such as ours that impede optimization of evidence-based navigation [48]. Our findings are therefore critical and timely from a policy perspective.

## Conclusions

This study observed high rates of acceptability of social needs screening on the part of patients and patient navigators; however, despite resources and training to achieve this health equity measure, the screening delivery rate was modest. We identify systems-level interventions that could enhance performance of social needs assessment, including integration of the navigator or other staff conducting the screening into the health care team, conducting screening during in-person encounters, employing screening with language and cultural congruity with the patient populations, and empathic training to increase efficacy in delivering social needs screening.

### Supplementary Information

Supplementary Material 1.

Supplementary Material 2.

## Data Availability

The quantitative datasets used and/or analyzed during this study are available from the senior author (T. Battaglia) upon reasonable request. Qualitative datasets will not be made available because doing so violates the study’s informed consent.

## Abbreviations

aRR: Adjusted rate ratio
CI: Confidence interval
RR: Rate ratio
TRIP: Translating Research Into Practice
