# Supplementary material for: Implementation of social needs screening for minoritized patients newly diagnosed with breast cancer: a mixed methods evaluation in a pragmatic patient navigation trial
Source: BMC Health Serv Res. 2024 Jul 9;24:783. doi: 10.1186/s12913-024-11213-7 (PMC11234663; doi:10.1186/s12913-024-11213-7)
Supplement: Supplementary file 1 — Supplementary Material 1. [file 12913_2024_11213_MOESM1_ESM.docx]

# TRIP STUDY: Follow-up Interview Guide-Implementation Outcomes

#

*Please note: This is an interview guide, intended to be used flexibly, to allow for a conversational flow to the interview while covering the topics below. Prompts are included here as possible suggestions for elaboration if responses are short.*

*Framing: The overall purpose of this interview will be to ascertain how the TRIP program has worked/been working at each site, for however long TRIP activities have been on-going. Questions should be phrased accordingly, depending on site’s roll-out date for TRIP.*

**INTRODUCTION**

Thank you for talking to me today. I want to learn about your experience with the Translating Research Into Practice (TRIP) study overall, as well as your opinions on specific components of TRIP.

Before we begin, I will provide a little information so that we have a shared understanding.

- *When we refer to TRIP, we mean the multi-site research study taking place across 5 Boston hospitals,to implement an enhanced Breast Cancer Patient Navigation standad of care.*
- *As a reminder, TRIP has been active at your site since [MONTH AND YEAR].*

There are no right or wrong answers to the questions I’m going to ask you today, I really just want to hear your thoughts and opinions.

Do you have any questions before we get started?

**PART 1: BACKGROUND, GENERAL KNOWLEDGE AND OPINIONS OF TRIP OVERALL**

**OPENING QUESTION:** **[navigator; clinical champion; supervisor]** To start, what is your title and position and how long have you worked at this hospital?

**[non-TRIP key personnel**]: To start, what is your title and position and how long have you worked at this hospital? What role do you play in the care of breast cancer patients at your institution? Have you heard of Translating Research into Practice, or TRIP, before today?

**Now I’d like to ask a few questions about TRIP generally.**

| **Concept** | **Questions** | **Prompts** |
| --- | --- | --- |
| Intervention Purpose | **[Navigators]** In your own words, what do you think the TRIP study is aiming to achieve? |  |
|  | **[Clinical Champions/Supervisors]** In your own words, what do you think the TRIP study is aiming to achieve? |  |
| Intervention Activities | **[Navigators]** In your own words, describe what activities you do that are specifically part of the TRIP intervention |  |
|  | **[Clinical Champions/Supervisors]** In your own words, describe what activities your navigation team does that are specifically part of the TRIP intervention. |  |

**PART 2: FIDELITY**

| **Concept** | **Questions** | **Prompts** |
| --- | --- | --- |
| **TRIP Fidelity: Navigation Guidelines/11 Steps** | **[Navigators]** Can you describe to what extent you were able to integrate the TRIP navigation guidelines into your navigation activities?  What about the guidelines worked for you? What about the guidelines didn’t work for you?  *Note for interviewers: focus on the steps that the interviewee brings up. No need to cover each of the 11 steps.* | 11 Steps (*show guidelines*)  What helped you implement the TRIP navigation guidelines? How/why?  What has impeded your ability to implement the TRIP navigation guidelines? How/Why?  What were your greatest successes? What were your greatest challenges? |
|  | **[Clinical Champions/Supervisors]** Can you describe the extent to which the TRIP navigation guidelines were integrated into the navigation program?  What about the guidelines worked within the navigation program? What about the guidelines didn’t work? | 11 Steps (*show guidelines*)  What were the greatest successes?  What were the greatest challenges? |
| **TRIP Fidelity: Managing Case Load**  **TRIP Fidelity: Communication across sites**  **TRIP Fidelity: Social Needs Screening** | **[Navigators]** How were you able to manage your TRIP case load?  Which tool did you use to accomplish this?  How were you able to communicate across sites to facilitate warm hand offs and patients transferring their care to other sites?  Which tool did you use to accomplish this?  How were you able to conduct a social needs screening with your TRIP patients?  Which tool did you use to accomplish this? | Can you give me an example of a time when you used this tool?  What made this work easier? What made this work harder?  Tell me about a time when you worked aross sites to facilitate a warm hand off or transfer a patient. What tools did you use? What made this work easier? What made this work harder?  Can you give me an example of a time when you used this tool?  How did this tool help you support your patients?  What made this work easier? What made this work harder? |
| **TRIP Fidelity: Managing Case Load**  **TRIP Fidelity: Communication across sites**  **TRIP Fidelity: Social Needs Screening** | **[Clinical Champions/Supervisors]**  To what extent were the TRIP navigators able to manage their case loads?  Which tool were used to accomplish this?  What made this work easier? What made this work harder?  To what extent were the navigators able to communicate across sites?  Which tools were used to accomplish this?  What made this work easier? What made this work harder?  To what extent were the navigators able to conduct a social needs screening?  Which tools were used to accomplish this?  What made this work easier? What made this work harder? |  |

**PART 3: ACCEPTABILITY**

**Now I would like to hear your opinions about the study – your likes, dislikes, and if TRIP a good fit for [*site*].**

| **Concept** | **Questions** | **Prompts** |
| --- | --- | --- |
| TRIP Overall Acceptability | **[Navigators]** What do you like about the TRIP overall?  What would you change about TRIP to support your day to day work? | Navigation guidelines?  Shared Registry?  Social Needs Assessment? |
|  | **[Clinical Champions/Supervisors]** What do you like about TRIP overall?  What do you dislike about TRIP?  What would you change about TRIP to support your day to day work? | Navigation guidelines?  Shared Registry?  Social Needs Assessment? |

**PART 4: ADOPTION *(research study ending, are you adopting the intervention)* and SUSTAINABILITY *(maintenance and sustainability in the future)***

| **Concept** | **Questions** | **Prompts** |
| --- | --- | --- |
| **TRIP Adoption** | **[Navigators]** When the study is over, what components of TRIP do you intend to use in your routine practice?  What would you need to sustain this? | How will you continue to screen for social needs and refer patients to resources when the study is over?  How will you continue to communicate across sites when the study is over?  How will you continue to manage your caseload when the study is over? |
|  | **[Clinical Champions/Supervisors** When the study is over, what components of TRIP do you intend to continue to use in your navigation program?  What would you need to sustain this? |  |

**PART 5: REACH/PENETRATION – to what extent is the TRIP approach spread throughout the institution**

| TRIP Penetration of navigation program w/in site context | **[Navigator]** To what extent do you feel like you are part of the larger cancer care team? How has TRIP changed this?  Thinking about the navigation guidelines (11 steps), who else on the cancer care team is involved in this navigation work?  Which other members of the navigation team use these guidelines? Or are responsible for some of these activities? | **Cancer Care Team** defined as: anyone who cares for breast cancer patients from diagnosis through survivorship |
| --- | --- | --- |
|  | **[Clinical Champions/Supervisors]** To what extent are the TRIP navigators part of the cancer care team?  How has TRIP changed this? | **Cancer Care Team** defined as: anyone who cares for breast cancer patients from diagnosis through survivorship  Tell me about a time when care integration worked well at your site. |

**PART 6: TRIP Training and Communication**

| TRIP Learning Climate | Since we’ve been working with you, the TRIP team has been communicating through: *monthly newsletters, weekly site email updates, quarterly TRIP Patient Navigator Network Meetings, and using REDCap messenger.*  How has your experience been with the TRIP trainings and support been?  How has your experience been with communications from the TRIP research team? | What went well? What could have been better?  Tell me about a time this communication worked well for you or your team?  Other examples of communication? |
| --- | --- | --- |
|  | **[Clinical Champions/Supervisors]** How has your experience been with the TRIP trainings and support?  How has your experience been with communications from the TRIP research team? | What went well? What could have been better?  *Newsletters? Weekly site emails? Patient Navigator Network Sessions?* |

Is there anything else you’d like to share about your experiences with TRIP?

**CONCLUSION**

Do you have any final questions?

Thank you for taking the time to speak with me about your experiences with TRIP.

- *Note to Interviewer: Turn off recorder at this point -*

**PART 7: DEMOGRAPHICS**

Before we end our time today, TRIP would like to learn a little more about your background.

We are asking all participants to complete this 1-page survey. It will ask about basic information like race and education.

*Interivewer to administer survey via REDCap at time of interview. Does NOT need to be recorded on audio.*
