# Supplementary material for: Implementation of social needs screening for minoritized patients newly diagnosed with breast cancer: a mixed methods evaluation in a pragmatic patient navigation trial
Source: BMC Health Serv Res. 2024 Jul 9;24:783. doi: 10.1186/s12913-024-11213-7 (PMC11234663; doi:10.1186/s12913-024-11213-7)
Supplement: Supplementary file 2 — Supplementary Material 2. [file 12913_2024_11213_MOESM2_ESM.docx]

## **TRIP Study**

## **Interview Guide - Patient Interviews**

**TRIP Operational Definition of Acceptability:**

**Goal**: To understand if patients were asked screened for health-related social needs, provided resources, and tracked over time to ensure identified needs were addressed/resources provided.

**Introduction:**

During your cancer journey, you’ve been provided navigation services by a number of individuals (nurses, SWs, PNs). These are services that are offered by the hospital that we need your advice on how to improve. During a person’s cancer journey, many things may come up that make it hard to get treatment. Some examples of this may include not having transportation to your appointments, having trouble paying rent, not being able to buy enough food, or not being able to pay bills. We’d like to understand your experience getting support around some of those things.

1. **During your care you were asked at least once a series of questions about these types of needs. Can you tell us what you thought about this? How did you feel when a member of your care team asked you about these needs? Did you find it helpful?**
2. **Can you briefly tell me whether you had transportation related needs during your cancer journey? (Yes/no)** *Probe if necessary: how did you get to appointments (private car, uber/lyft, public transportation), difficulty/ease of getting transportation, difficulty/ease of affording transportation, did you miss appointments or were you late due to transportation?*
   1. **Can you tell me whether or not your cancer team asked about any transportation related needs that might have come up for you during your treatment?**
      1. **If YES:** Can you tell me about your experience? How comfortable were you discussing your transportation related needs with your team? How did it make you feel receiving this support from your care team?
      2. **If NO:** How would you have felt if they had asked you? Would you feel comfortable discussing your transportation needs with your care team?
3. **Can you tell me about any housing related needs during your cancer journey?** *Probe if necessary: did you have difficulty paying rent/mortgage, did you have stable housing or have to move, did you feel comfortable and safe in your home?*
   1. **Can you tell me whether or not your cancer team asked about any housing related needs that might have come up for you during your treatment?**
      1. **If YES:** How comfortable were you discussing your transportation related needs with your team? How did it make you feel receiving this support from your care team?
      2. **If NO:** How would you have felt if they had asked you? Would you feel comfortable discussing your housing needs with your care team?
4. **Can you tell me about any food related needs during your cancer journey?** *Probe if necessary: difficulty/ease of accessing the food you needed, difficulty/ease of affording food, did you run out of food or skip meals?*
   1. **Can you tell me whether or not your cancer team asked about any food related needs that might have come up for you during your treatment?**
      1. **If YES:** How comfortable were you discussing your food related needs with your team? How did it make you feel receiving this support from your care team?
      2. **If NO:** How would you have felt if they had asked you? Would you feel comfortable discussing your food needs with your care team?
5. **Can you tell me about any financial or employment related needs during your cancer journey?** *Probe if necessary: did you lose a job, have reduced income or hours, have trouble paying bills or for treatment/medication, difficulty/ease affording basic needs, have to borrow money?*
   1. **Can you tell me whether or not your cancer team asked about any financial or employment related needs that might have come up for you during your treatment?**
      1. **If YES:** How comfortable were you discussing your financial or employment related needs with your team? How did it make you feel receiving this support from your care team?
      2. **If NO:** How would you have felt if they had asked you? Would you feel comfortable discussing your financial or employment needs with your care team?
6. **Do you feel your non-treatment needs were met by your care team?**
   1. Why or why not?
7. **Do you have any additional suggestions as to how hospitals can help cancer patients with these types of needs during their treatment?**
